# Entrustable professional activity use in emergency medicine: A scoping review

**DOI:** 10.1002/aet2.70035

**Published:** 2025-04-09

**Authors:** Tim Baker, Hannah Beks, Franco Schreve, Mary Lawson, Vincent L. Versace

**Affiliations:** ^1^ Centre for Rural Emergency Medicine School of Medicine, Deakin University Warrnambool Victoria Australia; ^2^ Deakin Rural Health School of Medicine, Deakin University Warrnambool Victoria Australia; ^3^ Western Hospital Footscray Victoria Australia; ^4^ School of Medicine Deakin University Geelong Australia

**Keywords:** competency‐based education education, graduate emergency service, hospital, medical

## Abstract

**Objective:**

The objective was to scope the literature and describe the extent and type of evidence about entrustable professional activities (EPAs) in postgraduate emergency medicine (EM) education.

**Methods:**

Joanna Briggs Institute's methodology was used to find and extract relevant data from documents found in Ovid MEDLINE, EMBASE, and CINAHL, supplemented by a gray literature search using Google Advanced for EPA frameworks. Eligible documents discussed EPAs for doctors in structured EM training programs. Data extracted included research methods, research approach, participants, scope, EPA element addressed, and dominant logic used by EPA creators.

**Results:**

Data were extracted from 58 documents. Thirty‐four of the documents (58.6%) were peer‐reviewed journal articles, 18 (31.1%) were conference abstracts, and six (10.4%) were curriculum documents from EM organizations. Thirty documents were from Canada (51.7%). Twenty‐five documents (43.1%) took an explorative approach. Twenty‐one documents (36.2%) were translational in approach. Thirteen EPA frameworks, containing a total of 158 EPAs, were found.

**Conclusions:**

EM is an expanding area of EPA development, but frameworks remain highly variable and unstandardized. Most studies are explorative or translational, leaving gaps in experimental research to justify EPA adoption and observational research to assess real‐world outcomes.

## INTRODUCTION

Emergency medicine (EM) educators must prepare graduates for independent practice that meets societal expectations for safe and effective emergency care.^1^, ^2^ However, defining the scope of practice for EM trainees is challenging, as the specialty encompasses a vast range of patient presentations, clinical procedures, and responsibilities essential to the delivery of emergency care.^3^ Traditional competency‐based education frameworks struggle to capture the breadth of EM practice in a way that is both comprehensive and practical.^4^ Entrustable professional activities (EPAs) can help by defining the core professional tasks that emergency physicians must perform independently in the workplace.^5^

Competence is a multidimensional and dynamic array of abilities across multiple domains, which varies with context, experience, and stage of training.^1^, ^6^ In contrast, a competency is an observable ability integrating knowledge, skills, values, and attitudes.^1^, ^6^ Typical competencies include collaboration, scholarship, and medical expertise.^7^ These competencies work together to develop competence over time. To use a musical metaphor, competence is like a symphony by a famous orchestra, where multiple elements come together at a specific time and place to create a cohesive performance, while competencies are like the orchestra's sections, each contributing sound that supports the whole.

However, while competencies describe individual abilities, they do not inherently define how these abilities translate into real‐world clinical practice.^8^ EPAs complement competencies by focusing on how well trainees perform key workplace tasks.^8^ ten Cate et al.^9^ define EPAs as “a unit of professional practice that can be fully entrusted to a trainee, once he or she has demonstrated the necessary competence to execute this activity unsupervised.” The key distinction is that competencies describe the qualities of the person^10^ while EPAs describe the work. Each EPA is like a musical piece in an orchestra's repertoire.

An EPA is a typical task that somebody must complete.^8^ Clinicians can struggle to assess a person's competence by directly translating what they see them do in the workplace into competencies.^11^ EPAs make it easier for a supervisor to assess a person's competence by deciding how much supervision the junior doctor will need to perform each relevant task (EPA) safely.^8^ Senior doctors make this kind of workplace decision in clinical practice most days.^8^, ^12^ Returning to the metaphor, an artistic director may find it more intuitive to evaluate the quality (competence) of an orchestra by critiquing its performance on several key pieces of music (EPAs) rather than attempting to assess the individual contributions of the string, woodwind, and percussion sections (competencies). Similarly, an emergency physician evaluating the competence of a senior trainee may find it more practical to assess how well they perform critical care procedures and manage geriatric patients (EPAs) than directly appraising their collaboration, scholarship, and medical expertise (competencies).

The most valuable EPAs are authentic steps on the path to becoming a specialist.^5^ When a doctor becomes proficient at a particular EPA, it should signify that they are ready for a new level of autonomy.^13^ Doctors from different specialties mostly require the same competencies—medical expertise, collaboration, and professionalism.^13^ However, the tasks they undertake each day are very different—“initiating antipsychotic medication in a patient with schizophrenia,”^14^ “performing an appendectomy,”^8^ or managing a complicated birth.^13^ Specialties have, therefore, developed their own EPAs.^5^ These EPAs can outline the minimum set of professional activities a new specialist should be able to perform independently on their first day.^5^

EM has been noted to be slower than surgery, pediatrics, and internal medicine in introducing EPA frameworks,^15^, ^16^ but EPA development in EM has reached a crucial phase. The first generation of EPA frameworks have been created for EM.^15^, ^16^ Significantly, in 2018, the Royal College of Physicians and Surgeons of Canada added 28 EPAs to their nationwide competency‐based EM education program.^2^ Early studies evaluating elements of this program^17^ and suggesting modifications^18^ are now available. A review of EPAs in undergraduate and foundation‐year EM has been published.^19^

Although EPA frameworks are usually created by consensus decision‐making methods, valid EPA frameworks still require a robust underlying evidence base.^20^ In one study, 31% of medical specialty EPA creation teams reviewed the literature as a first step.^16^ Reviewing the literature is also helpful for educators who have already introduced an EPA framework. While early studies focused on EPA creation,^21^ researchers are now examining issues such as improving teacher–learner interactions at the micro level, strengthening institutional programs by technology and other means at the meso level, and linking EPAs across disciplines and professions at the macro level.^21^

A scoping review is helpful for mapping areas where the heterogeneous evidence base usually precludes a critical appraisal.^22^, ^23^, ^24^ Other postgraduate specialties have completed EPA scoping reviews,^15^, ^25^, ^26^ and there is a scoping review for undergraduate EM EPAs.^19^ A preliminary search of MEDLINE was conducted, and no scoping reviews of EPAs for postgraduate EM were identified. The objective of this review is to scope the literature for evidence of the use of EPAs in the EM context, including the development and modification of EPA frameworks to educate and assess trainees.

### Review questions

1. How have EPAs for post–foundation‐year EM been evaluated and discussed in the international literature?
2. What are the characteristics of EPA frameworks created for post–foundation‐year EM in the international literature?

## METHODS

This review follows the Joanna Briggs Institute (JBI) scoping review methodology.^22^ The protocol was published before the study began (https://osf.io/n2hkd/).

### Inclusion and exclusion criteria

JBI suggests that scoping review research questions, search terms, and inclusion and exclusion be based on participants, concepts, and context (see Table S1). The participants were doctors who were training and working specifically in EM. Most of these doctors will be training to be EM specialists. However, in rural areas, many emergency departments (EDs) are staffed by doctors who have gained their EM skills through family medicine or generalist medicine training.^27^ These trainees may undertake rotations in EDs to prepare to work some or all of their careers in EM.^28^, ^29^ EPA frameworks for these rotations were considered to be in scope.

This study excludes medical students and junior doctors in their foundation years.^30^ In this study, foundation‐year doctors are defined as junior doctors (typically in PGY‐1 or PGY‐2) who have not yet entered structured specialty training—a pathway used in the United Kingdom, Australia, Denmark, Sweden, Israel, and Japan.^31^ EPAs for these doctors are designed to improve emergency care skills broadly across all specialties (for example, managing a patient with syncope in a clinic), rather than to prepare doctors for the specific work of EM. This distinction aligns with Passoni Lopes et al.,^19^ who reviewed EPAs for medical students and early junior doctors not yet in specialty training. Conversely, this study includes PGY‐1 doctors from countries such as the United States and Canada,^31^ where trainees enter a structured EM training program immediately after medical school. EPAs that trainees from other medical specialties may perform while in the ED, such as a pacemaker check by a cardiology trainee, are not designed for EM trainees and were excluded.

The concept was EPAs. This term is easily searchable as the word entrustable is a neologism^9^—defined as a newly coined nondictionary word that has gained widespread acceptance. Although authors do not always adhere to the definition suggested by ten Cate et al.,^8^ variations appear to occur within the term EPA^9^ rather than with new names for similar concepts.^32^

The context is EM rather than an ED. EM includes aspects of prehospital retrieval and disaster medicine.^3^ EPAs in these areas contribute to our objective. Emergency trainees may complete EPAs while rotated to other hospital areas (like completing airway tasks in an anesthesiology rotation). Studies addressing these situations were considered to be in scope.

### Search strategy

A scholarly services librarian assisted in the creation of a peer‐review search strategy (see 2S in the supplement) that was run in Ovid MEDLINE ALL, EMBASE, and CINAHL databases on May 1, 2024, with no date limit. All article types, including experimental and quasi‐experimental study designs, review articles, and opinion papers, were accepted. Foreign language studies were accepted and translated. SCOPUS was used to identify relevant references and citations.

The gray literature search strategy was run to identify relevant theses in Open Access Theses and Dissertations and ProQuest Dissertations & Theses Global. Google Advanced was searched for portable document format items containing the terms “entrustable professional” and “emergency.” This is a modification of the search outlined in the protocol, which used “entrustable” rather than “entrustable professional.” The change was made due to the predominance of unrelated articles with the initially suggested term. A limit of 1000 results was chosen, consistent with previous scoping reviews.^33^ Haddaway et al.^33^ note that most relevant citations occur in the first few hundred results, further supporting this approach. A focused search of the websites of the International Federation of Emergency Medicine and its member organizations was then employed using the above terms as well as known foreign language versions of the term EPA (see Table S3).

### Document selection

The title and abstract of documents identified by the search strategy were uploaded into Covidence systematic review software (Veritas Health Innovation), and duplicates were removed. Two reviewers (TB and HB) independently screened document titles and abstracts, sorting documents into included, excluded, and maybe groups (see Table S1). Full texts of the included and maybe group articles were downloaded. The same reviewers assessed these documents and recorded reasons for any exclusions. Disagreements between reviewers were resolved through discussion.

### Data extraction and analysis

Two reviewers (TB and HB) extracted data from 10% of the included articles to assess the appropriateness of the data extraction tool and clarify definitions. One reviewer then extracted data from all included articles using this tool (see Data S2–Data Extraction Tool). Classifications for the article approach were adapted from The Research Compass by Ringsted et al.^34^ Classifications of EPAs as procedure based, disease/patient group based, and service provision based were adapted from the design logic method of Hennus et al.^35^ Service provision EPAs are essential responsibilities that can be assigned to a clinician for completion but are not tied to a particular procedure or patient group.^35^ These tasks support the safe and efficient functioning of the ED and include coordinating transitions of care, managing departmental flow, and supervising junior staff.

Data were collected from the introductory and methods sections and supplementary appendices if they contained a description of EPAs. Data from the results sections of studies were not presented. This aligns with the JBI scoping review methodology^22^ that states that scoping reviews be used as a first step to map the evidence available where the literature is too heterogeneous (as thought likely to be in this case) to allow a useful comparison of results or an understanding of their validity, even if a critical appraisal was undertaken.

Extracted data were exported from Covidence systematic review software to Microsoft Excel (Version 16.91, Microsoft Corp.) for data organization and cleaning. Analysis was performed using Microsoft Excel and IBM SPSS Statistics (Version 29.0, IBM Corp.). Findings were synthesized and described using narrative synthesis following scoping review methodological guidelines.^22^

## RESULTS

### Document identification

**FIGURE 1:**
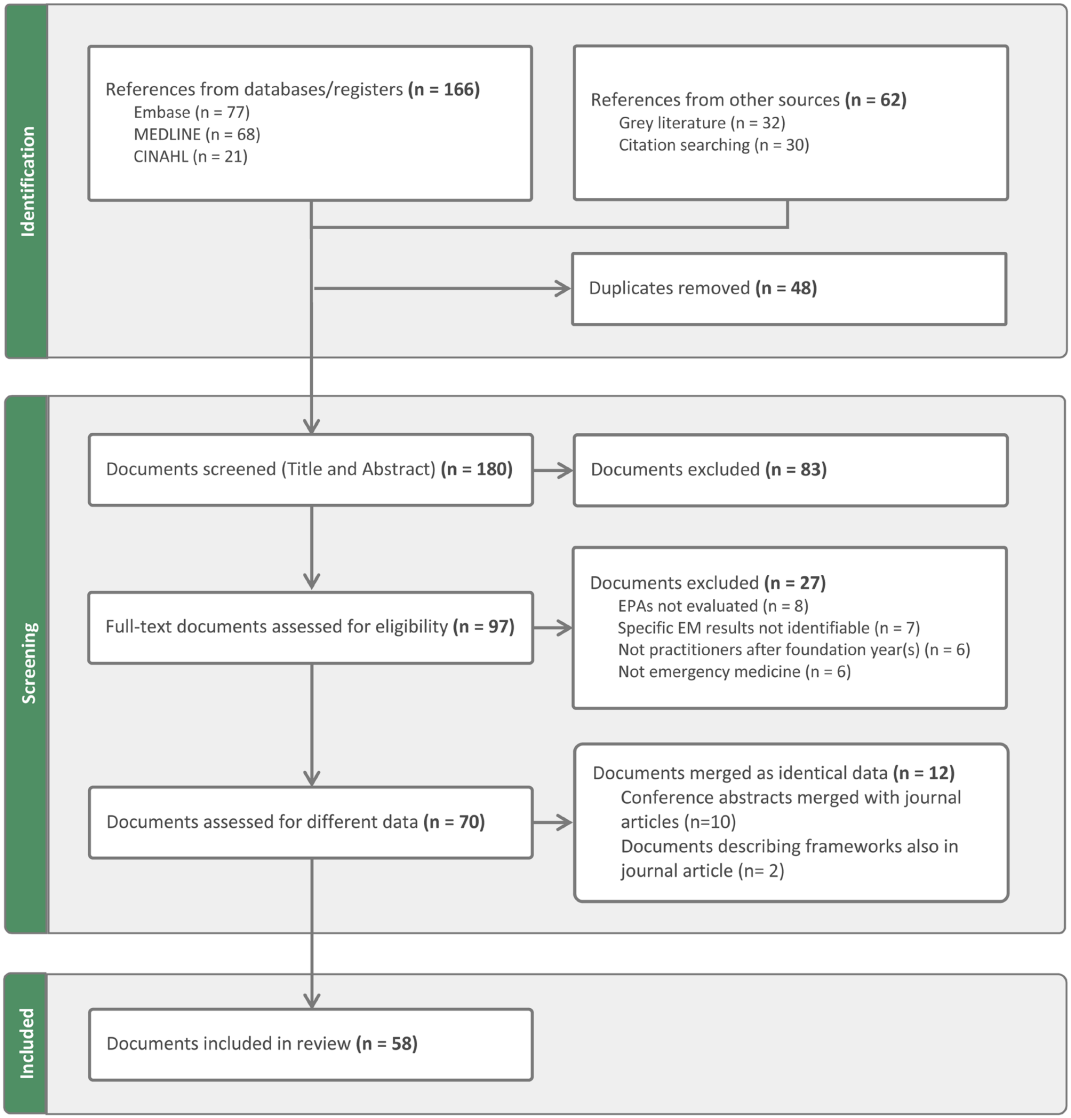
PRISMA scoping review flowchart. This chart outlines document identification, selection, and screening according to the search strategy and inclusion and exclusion criteria of the scoping review of EPAs for EM. It displays them according to the PRISMA ScR format.^36^ EPAs, entrustable professional activities.

The database search strategy identified 166 articles, supplemented by 62 records from the gray literature and citation searches (see Figure 1). After duplicate removal and screening, 58 documents were included (see Table 1).

**TABLE 1:** Aims and methods of included studies.

Identifier	Aim/purpose	Specific methods	Topic focus
Aberdour 2017^37^	To create EPAs for stroke management by emergency and internal medicine trainees	Modified Delphi process	Stroke management
Akbar 2024^38^	To construct a learning management system for EPA assessment	Descriptive case study	General EM
Alrimawi 2020^39^	To assess whether case‐based learning helps trainees recognize EPA‐suitable cases and increases EPA completion	Pretest‐posttest comparison group design	General EM
Beeson 2014A^40^	To create EPAs and map them to milestones	Survey‐based consensus building	General EM
Beeson 2014B^41^	To describe EPAs and how they relate to EM milestones	Review article	General EM
Bérczi 2020^42^	To design an addiction medicine rotation curriculum based on EM EPAs	Learning needs assessment	Addiction medicine
Borman‐Shoap 2018^43^	To assess if reminder notices increase the rate of EPA assessments performed and to assess barriers to EPA completion	Pretest‐post comparison groups/Questionnaire	Pediatric EM
Boyne 2024^44^	To implement a novel EPA‐based end‐of‐shift assessment	Organizational change management research	General EM
Breckwoldt 2022^45^	To explore lifelong learning in EM using EPAs	Review article	General EM
Caretta‐Weyer 2024^46^	To create EPAs for EM	Modified Delphi process	General EM
Carey 2020^47^	To develop a learning management system that meets trainee needs	Grounded theory with implementation component	General EM
CEP Thailand 2019^48^	To outline an EM training curriculum	Curriculum document	General EM
Chan 2020^49^	To define a list of educational and clinical outcomes for EPA‐based EM training programs	Divergent convergent consensus process	General EM
Chang 2023^50^	To identify the association between milestones and EPAs with professional identity	Cross‐sectional study	General EM
Collings 2019^51^	To compare anonymous supervisor assessments with workplace‐based assessments	Psychometric study	General EM
Costello 2019^52^	To create a curriculum to improve trainee performance for an EPA on managing unstable patients	Kerns six‐step approach to curriculum development	Resuscitation in EM
Costello 2023^53^	To explore trainee perceptions of simulation for EPA assessment	Grounded theory	General EM
Denson 2013^54^	To identify and address gaps in team‐based learning using EPAs	Mixed methods needs assessment	Geriatric EM
Deutscher 2021^55^	To design a curriculum addressing an EPA on patients at risk of violence and neglect	Kerns six‐step approach to curriculum development	Human trafficking
Fant 2015^56^	To develop an EM orientation curriculum based on EPAs and map them to milestones	Curriculum evaluation research	General EM
Fisk 2023^57^	To explore supervisor perceptions of simulation for EPA assessment	Qualitative semi‐structured interviews	General EM
Golden 2021^58^	To assess EPA alignment with quality standards using the EQual rubric	Descriptive study using a validated tool	General EM
Hall 2020^59^	To evaluate the experience of residents and supervisors as an EPA‐based program was introduced	Case‐study methodology using iterative cycles	General EM
Hart 2019^4^	To describe the development of milestone‐linked EPAs for EM	Glaser's state‐of‐the art approach to consensus	General EM
Hsiao 2020^60^	To develop a learning management system for EPA‐based learning and assessment	Formative implementation research	General EM
Hsu 2016^61^	To describe the development of EPAs for pediatric EM	Consensus methodology	Pediatric EM
Hsu 2023^62^	To determine supervisors' view of the level of entrustment required to pass and practice in Pediatric EM	Cross‐sectional survey	Pediatric EM
Jaber 2024^63^	To examine the validity of an end‐of‐shift EPA assessment	Psychometric study	General EM
Koh 2019^64^	To map ED interventions required to meet EPAs associated with opioid use patients	Literature review	Addiction medicine
Lai 2019^65^	To assess time‐based increases in autonomy in preparation for EPAs	Quantitative survey	General EM
Landreville 2022^66^	To determine if direct or indirect observation influences the quality of EPA assessment	Cross‐sectional study	General EM
Lee 2020^67^	To explore the impact of a new online EPA assessment platform	Description of user experiences	General EM
Lee 2021^68^	To explore the quality of EPA feedback	Analysis of narrative feedback against desired model	General EM
Lui 2023^69^	To outline a competency‐oriented workplace‐based assessment	Curriculum document	General EM
Pandya 2022^70^	To trial the use of simulation for resuscitation EPAs	Implementation followed by survey‐based program evaluation	Resuscitation in EM
Paterson 2023^71^	To identify barriers and facilitators to acquiring high‐quality EPA assessments	Qualitative framework analysis	General EM
Prudhomme 2020^72^	To assess the validity of EPAs in simulated and real‐world settings	Prospective cohort study	Resuscitation in EM
RCPSC 2017^73^	To describe an EM training program	Curriculum document	General EM
Sagalowsky 2023^74^	To summarize revisions to the pediatric EM curriculum	Explanation of guideline	Pediatric EM
Sahi 2024^75^	To evaluate the current use of simulation for EPA assessment	Mixed methods	General EM
Sample 2021^76^	To codesign and evaluate interventions to improve EPA acquisition	Implementation study	General EM
Saraburi 2018^77^	To describe an EM training program	Curriculum document	General EM
Seed 2023^78^	To assess simulation‐based assessment for resuscitation‐focused EPA	Mixed methods	Resuscitation in EM
Sherbino 2020^2^	To describe the creation of a new curriculum	Description of creation of a new curriculum	General EM
Singh 2023^79^	To determine the influence of longitudinal coaching relationships on the quality of EPA assessments	Cohort study	General EM
Spadafore 2024^80^	To describe how natural language processing and machine learning was used to predict the quality of EPA feedback	Description of implementation and operation of process	General EM
Stanford PEM 2016**	To outline a Pediatric EM fellowship program	Curriculum document	Pediatric EM
Stoneham 2019^81^	To describe the creation of EPA reference cards for trainees	Implementation study	General EM
Taiwan SEM 2019^82^	To implement a curriculum for EM	Curriculum document	General EM
Thoma 2020^83^	To assess the number of EPAs per resident and time to resident promotion	Secondary analysis EPA program data	General EM
Tiyyagura 2014^84^	To understand how factors (including simulation) affect entrustment decisions	Grounded theory using semi‐structured interviews	Pediatric EM
Turner 2021^85^	To investigate program directors' perceptions of the minimum level of supervision required for a trainee to graduate	Cross‐sectional study	Pediatric EM
Villa 2024^86^	To develop EPAs for EM Education Fellows	Delphi method	Education in EM
Vincent 2023^87^	To develop a digital infographic to improve EPA acquisition	Needs assessment	General EM
Woods 2022^88^	To validate the QuAL score of EPA supervisor narrative comments by comparing with end‐user assessments of utility	Rating of de‐identified EPA narrative comments	General EM
Woods 2023^89^	To develop an NLP model for applying the QuAL score to supervisor narrative comments	Modelling to create a derivation and validation set	General EM
Yilmaz 2022^90^	To create a learning management system dashboard based to assist faculty development	Design‐based research based on thematic interviews	General EM
Yilmaz 2023^91^	To develop a learning management system dashboard suitable for program assessment	Design‐based research based on thematic interviews	General EM

Abbreviations: EPA, entrustable professional activity; NLP, natural language processing.

** Not in reference list. Reference is Pediatric Emergency Medicine fellowship program. Program manual pediatric emergency medicine 2017–18. Standford University; 2016. Accessed May 10, 2024. https://med.stanford.edu/content/dam/sm/emed/documents/fellowships/pem/PEM‐Manual.pdf.

### Document characteristics

**FIGURE 2:**
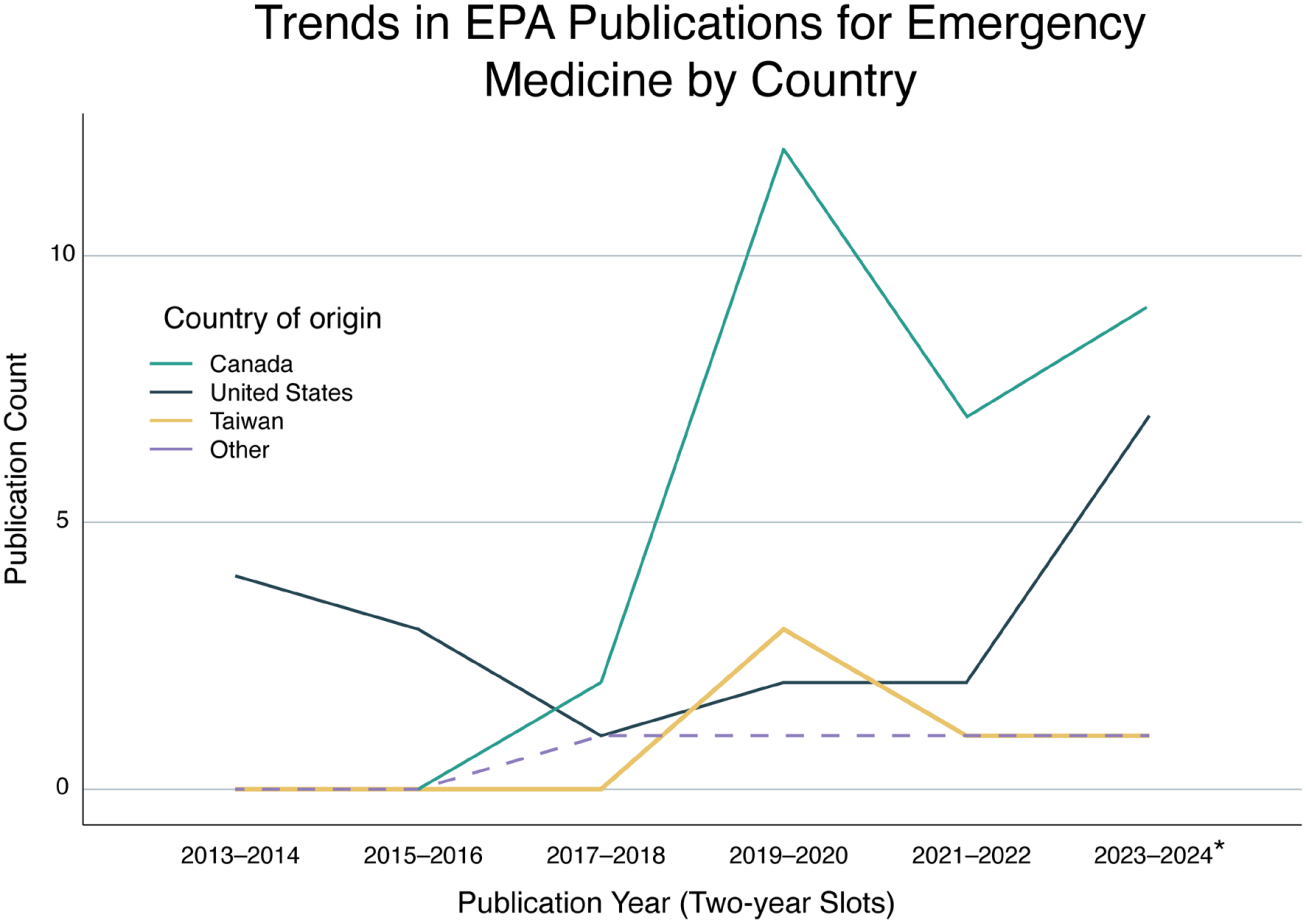
Publication Trends in EPAs for EM by country (2013–2024). This graph shows the number of publications (peer‐reviewed and gray literature) on EPAs in EM by country from 2013 to 2024, with counts grouped into 2‐year intervals. Publications outside of Canada, the United States, and Taiwan are grouped under “other” (Thailand two, Hong Kong one, Switzerland one). *Please note that data for the 2023–2024 interval are incomplete, as data collection concluded in May 2024. EPAs, entrustable professional activities.

The first EPA article was published in 2013.^54^ Relevant EPA documents were produced from six countries (Canada 30 [51.7%], United States 19 [32.8%], Taiwan five [8.6%], Thailand two [3.4%], Hong Kong one [1.7%], and Switzerland one [1.7%]). An upward trend in document creation was noted in Canada and the United States (Figure 2). Thirty‐four of the documents (58.6%) were published as peer‐reviewed journal articles, 18 (31.1%) were conference abstracts published in peer‐reviewed journals, and six (10.4%) were curriculum documents from EM organization websites describing EPA frameworks (see Table S4 for details).

Of the 52 documents published in journals, 10 (19.2%) included EPA titles, an additional 30 (57.7%) identified the EPA framework forming the basis of the study, and 12 (22.2%) did not identify a specific EPA framework. The EPA framework of the Royal College of Physicians and Surgeons of Canada was referred to by 28 articles (53.8%), and the framework from the American Board of Pediatrics was referred to by five articles (9.6%; see Table S4 for details of EPA frameworks).

### Focus

Forty‐one documents (70.7%) discussed EPAs covering general EM, seven (12.1%) discussed pediatric subspecialty EM, and one (1.7%) was for geriatric EM. Ten documents addressed EPAs for specific aspects of EM. These included four documents on resuscitation (6.9%); two documents on aspects of addiction medicine in EM (3.4%)^42^, ^64^; and one document each (1.7%) on EM aspects of stroke management,^37^ human trafficking,^55^ and medical education.^86^

All but one document concerned EPAs for post–foundation‐year doctors training in EM. Fifty‐one documents (87.9%) identified that the trainees were undertaking EM specialty training. Six documents (10.3%) did not identify the EM training pathway. No documents addressed EPAs for EM trainees from family medicine or rural generalist training. One article (1.7%) discussed EPAs as a component of lifelong learning for EM specialists but did not include EPA titles^45^ (see Table S4 supplemental for full details).

### Approach and elements

**FIGURE 3:**
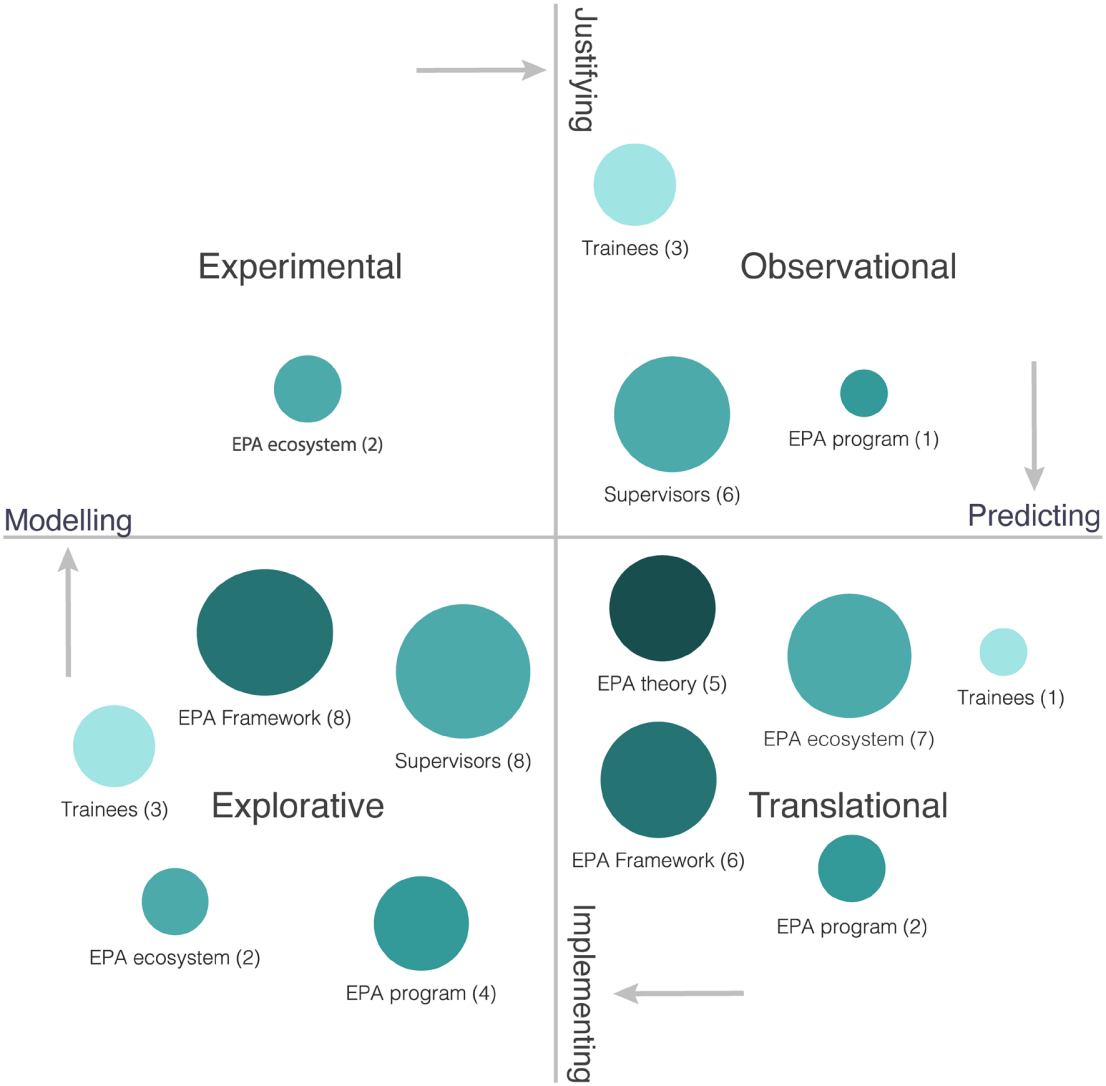
Mapping research on EPA for EM elements using the Research Compass framework. This chart presents publications (from both peer‐reviewed and gray literature) from 2013 to May 2024, mapped onto the four approaches outlined in the Research Compass framework^34^: explorative, experimental, observational, and translational. Each bubble represents research focusing on a specific element of the EPA system (each element has a unique color), with the size of the bubble corresponding to the number of publications. Notably, a significant portion of the research takes an explorative or translational approach to focus on an EPA framework. EPAs, entrustable professional activities.

The included documents had a wide range of aims and methods (see Table 1 and Table S4 for full details). To assist in the analysis, the aims and methods were grouped according to the four quadrants of the education research compass approach^34^ (Figure 3). Most documents were at either end of the research cycle: the explorative quadrant, which includes studies to assist idea modeling, contained 25 documents (43.1%), while the translational quadrant, which includes studies to aid implementation, contained 21 documents (36.2%). The more empirical quadrants in the middle of the cycle, used for justifying and predicting EPA components, contained fewer documents. There were two experimental studies (3.5%) and 10 observational studies (17.3%).

These results were further categorized by which EPA element they primarily addressed, spanning macroelements like EPA theory and frameworks, through mesoelements like specific hospital EPA programs and elements of the ecosystem (such as learning management system dashboards) to microelements discussing the attitudes and performance of supervisors and trainees. Fourteen documents (24.1%) discussed supervisors, 14 (24.1%) EPA frameworks, 11 (19%) ecosystem components, seven (12.1%) specific programs, seven (12.1%) trainees, and five (8.6%) EPA theories.

The 14 EPA framework documents are divided between eight explorative articles (13.8%) describing how frameworks were created (using Delphi and other consensus methods) and six translational documents (10.4%). These six were all curriculum documents created by EM organizations explaining how to implement EPAs into the curriculum. Supervisor assessments, entrustment decisions, and feedback were explored by eight documents (13.8%) using qualitative approaches and six documents (10.4%) using observational approaches. Seven documents (12.1%) assisted in translating EPAs into practice through ecosystem elements such as novel learning management systems, reference cards, infographics, and orientation programs. The only two studies (3.5%) in the experimental quadrant assessed whether elements of the ecosystem, such as case‐based orientation or reminder notices, increased the rate of EPA completion. Five literature reviews (8.6%) translated information from the competency‐based training literature for an education and clinical audience. Simulation for EPA assessment was the most common topic for documents focusing on trainees (two [3.5%] explorative, one [1.7%] observational, and one [1.7%] translational).

### Framework characteristics

**FIGURE 4:**
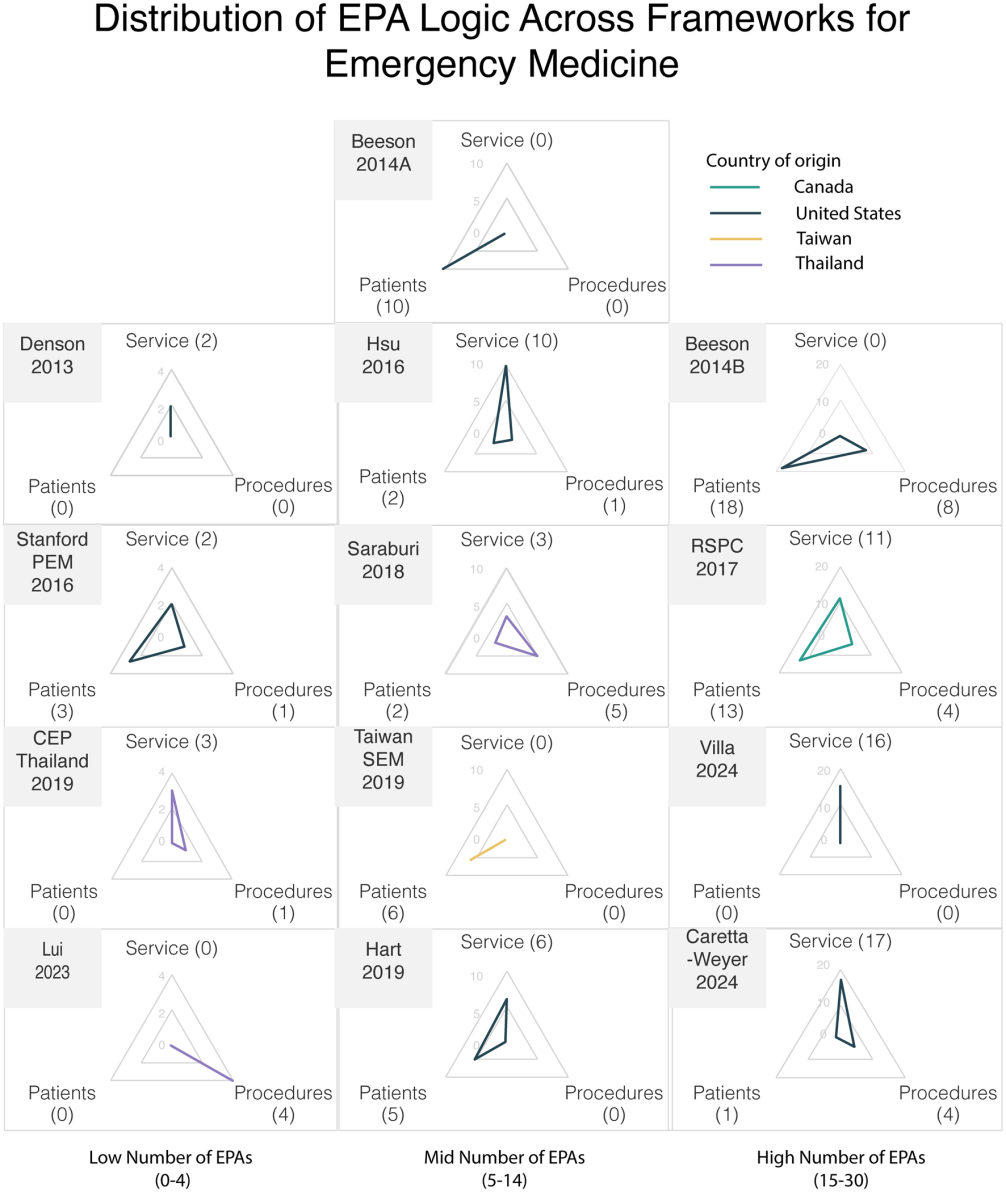
Distribution of EPA logic across EM frameworks, grouped by number of EPAs (low, mid, high). This chart illustrates how EPA frameworks vary in the number of EPAs based on the underlying logic approach of Hennus et al.^35^ of service provision, patient groups/diseases, and procedures, each element being an axis. All figures are the same size but represent different total numbers of EPAs. They are grouped into three columns by total EPA number: low (0–4), mid (5–15), and high (15–30). Refer to the data labels at the edges of each triangle for the exact EPA counts. More recent frameworks are lower, and color‐coded lines signify the country of origin of the framework (Canada, United States, Taiwan, and other) to highlight patterns occurring between countries and over time. EPAs, entrustable professional activities.

Thirteen EM EPA frameworks were identified from the 58 documents. While most articles came from Canada, only one framework (7.7%) did. Eight frameworks were from the United States (61.5%), two were from Thailand (15.4%), and one each (7.7%) was from Hong Kong and Taiwan (see Figure 4).

A total of 158 EPA titles were identified with a median of 10 EPAs per framework (range 2–28; see Table S5 for titles). The logic behind each EPA title was divided using the method described by Hennus et al.^35^ into EPAs based on service provision (70 EPAs, 44.3%), diseases/patient groups (60 EPAs, 38%), or procedures (28 procedures, 17.7%). The results for each framework are displayed as radar charts in Figure 4. Four frameworks (30.8%) use one logic type, four use two logic types (30.8%), and five use three logic types (38.5%). In six frameworks, service logic predominates; in five, patient groups/diseases predominate; and in two, procedures predominate.

There is no obvious association to be found between the year or country of publication and the ratios of EPA logic used (and displayed as differently shaped triangles on the radar charts). However, comparisons are difficult because some frameworks describe narrow EPAs while others group them into broad EPAs. For instance, the framework from Saburi Hospital in Thailand^77^ lists individual procedures such as rapid sequence intubation, central venous access, and procedural sedation. By contrast, the pediatric EM framework described by Hsu et al.^61^ lists a single procedural EP as “Demonstrate competence in performing common procedures associated with the practice of pediatric EM.” Differences between narrow and broad EPAs can also be seen for EPAs based on diseases and patient groups. Beeson et al.^41^ list 18 specific patient presentation EPAs (chest pain, depression, etc.), whereas Hart et al.^4^ group patient‐based EPAs under five titles, which are combinations of patient complexity, acuity, and stability.

## DISCUSSION

This scoping review found that educators and researchers in post–foundation‐year EM training increasingly share their knowledge about EPAs through journal articles, conference presentations, and curriculum documents. Findings indicate that most of the literature was produced in Canada, whereas most frameworks were developed in the United States. Authors of the literature included have written about all aspects of EPAs, from frameworks to specific online tools that simplify the recording of assessments. Teams have used open‐ended questions to explore stakeholder understanding and create EPA frameworks. Others have then translated what they know into practical advice on implementation. Some researchers have observed correlations between aspects of the EPA system, but few have been able to complete experimental studies. Thirteen EPA frameworks have produced 158 EPAs that outline the patient groups, procedures, and service requirements of EM.

Prior to recent developments, EPA experts have not considered postgraduate EM an active area for EPA research. Review articles in 2021^15^ and 2022^35^ found one EM EPA framework (compared to seven for surgery, five for pediatrics, and three for internal medicine).^15^ A 2018 literature search for EPA research in all areas of postgraduate medical education^16^ using similar sources found 49 research articles, with only three addressing EM. In contrast, our study found 11 EM frameworks created before 2021 and eight EM documents published before 2018. This suggests that earlier reviews may have underrepresented EM EPA frameworks due to differences in search strategies. In particular, the search strategy used for this review incorporated foreign language terms and targeted searches of EM professional organizations.

More recently, interest in EM EPAs has accelerated. The majority of research documents, not just frameworks but studies analyzing their development, application, and impact, have been published after the 2018–2022 reviews. A significant driver of this growth has been the 28 published documents citing the Royal College of Physicians and Surgeons of Canada EPA framework. Given its centrality to Canadian specialist EM training, EPAs may now be referenced in the EM education literature, even when the primary focus is on another aspect of training.

A novel aspect of this review was the use of the Research Compass^34^ to group the many different approaches taken by researchers. Using this system, over three‐quarters of the documents were in the explorative or translational quadrants. We found many documents describing how teams explored EPA concepts to model how EPAs work and create EPA frameworks. Other documents described how teams translated their EPA, education, and software knowledge into practical tools they could implement in their program. This predominance of explorative and translational studies is common in other areas of medical education research and is also reflective of most EPA research. A review by ten Cate et al.^21^ suggested that the majority of EPA research has focused on the development and early implementation of EPAs, with fewer studies examining educational or clinical outcomes. Another review^16^ using a classification similar to the research compass placed three‐quarters of the postgraduate EPA articles in a development group and one‐quarter in an implementation and assessment group. While explorative and translational research have shaped how EM EPAs are conceptualized and implemented, their real‐world impact on training and clinical outcomes has rarely been tested.

Observational studies have begun to address this gap, but experimental studies remain scarce. This review identified 10 observational studies that navigated the methodological challenges of complex variables and uncontrolled learning environments^34^ to assess EPAs in real‐world settings. However, only two experimental studies were identified.^39^, ^43^ This reflects broader challenges to experimental methods in medical education research, where it is difficult to link interventions to real‐world education and clinical outcomes.^21^, ^34^ The two experimental studies identified in this review used a simple outcome measure: the number of completed EPA evaluations.^39^, ^43^ Furthermore, the limited number of large‐scale EPA programs makes it challenging to measure meaningful effects with sufficient statistical power.^21^

Researchers examined the spectrum of EPA elements from large to small scale. We found research focusing at the microlevel on trainees and supervisors (in our review, articles on supervisors outnumbered those on trainees), at the mesolevel articles on technology to assist the operation of an EPA program and at the macrolevel documents on EPA frameworks. This aligns with ten Cate et al.,^21^ who suggest postgraduate EPAs should be researched at the micro‐, meso‐, and macroscales.

None of the EM frameworks exceeded the suggested maximum of 30 EPAs^10^ for a postgraduate training program, but nine had fewer than the recommended minimum of 20.^10^ The most common design logics for EPAs were service provision (44.3%) and diseases/patient groups (38%). This resembles EPA frameworks in general, where Hennus et al.^35^ found that EPAs were based on disease/patient groups (39%) and service provision (37%). Unlike other specialties that create EPAs for specific diseases,^35^ EM EPA creators have modified this EPA logic to describe patient presentations such as “shortness of breath”^41^ or “manage a low‐acuity, low‐complexity ‘stable’ patient.”^4^ Similarly, some frameworks have collated essential EM procedures into broader categories, such as basic procedural skills^73^ or critical care interventions.^48^ However, no consensus exists on which structure best aligns with EM training needs.

While scoping reviews do not assess research quality,^92^ they can identify areas lacking research. New experimental and observational research is essential for justifying EPA frameworks and predicting their impact, but further research is needed across all quadrants of the Research Compass. In addition, opportunities exist at the micro (individual), meso (institutional), and macro (framework and policy) levels.

In the explorative quadrant, macrolevel research could support an influential EM organization in establishing a core set of EPAs to strengthen global training while allowing for local adaptation. With 13 EPA frameworks now available, studies could compare models that incorporate different balances of procedural, patient‐type, and service EPAs. Beyond EPA titles, full EPA descriptions^9^—including specifications, limitations, context, and supervision levels—may help modify EPAs to reflect areas where general EM overlaps with pediatric and geriatric EM. They may also clarify broader emergency care roles where rural generalists and family medicine–trained physicians provide ED care.

To advance research in the experimental quadrant, at the microlevel, studies could evaluate how supervision models, feedback strategies, and assessment frequency influence the validated procedural outcomes recorded in clinical registries,^93^, ^94^ such as intubation success or complication rates. This approach would expand beyond upstream measures like EPA completion rates (used by the experimental research included in this review) to focus on clinically relevant outcomes. If small‐scale studies demonstrate meaningful impact, a multi‐institutional EPA research network could scale up the investigation at the macrolevel to assess whether implementing an EPA‐based training system leads to broader clinical improvements. Key challenges include achieving statistical power for low‐frequency outcomes and isolating EPA impacts from broader institutional variability.

In the observational quadrant at the macrolevel, a systematic review using the EQual rubric^95^ could assess how existing frameworks align with EPA standards. This scoping review has shown that there are sufficient frameworks and EPA titles to make such an appraisal feasible, but variability in their purpose and structure limits its utility. An exploratory approach, as described above, may be more effective in establishing a core EPA set. At the mesolevel, learning analytics using multi‐institutional data sets can assess how contextual factors, such as high‐ versus low‐volume centers, rural versus urban training, or differences in clinical exposure, affect not only trainee progression but also which EPAs are best supported by each context. Understanding these patterns may suggest whether certain environments facilitate earlier achievement of specific competencies.

In the translational quadrant, educators have applied natural language processing to tools like the QuAL score^96^ to assess and enhance supervisor feedback. At the micro‐ or mesolevels, research could support the implementation of artificial intelligence (AI)‐driven tools to refine real‐time feedback, improving specificity and usability while reducing faculty workload. Voice‐to‐text translation and automated structuring of narrative comments may further streamline EPA assessments, but even partial reliance on AI for entrustment decisions requires careful consideration. Taken together, these research opportunities highlight the need for a comprehensive approach to EPA development and evaluation across all levels and all quadrants of the Research Compass.

## LIMITATIONS

A scoping review was undertaken given the heterogeneity of the literature and insufficiency for a valid meta‐analysis with a critical appraisal. This limits us to describing the characteristics of studies and frameworks rather than assessing their quality^97^ (using tools such as EQal^95^). This also influences our section on design logic as the key article^35^ includes a critical appraisal component that places EPAs appraised as failing to meet the official definition of an EPA in an “other” category. Without critical appraisal, we could not do that.

We may have missed EPA documents behind firewalls or existing as webpages rather than portable format documents. The research compass^34^ provided us with a method to group articles to discern a broad pattern in the relevant literature, but it also has limitations. The definitions do not sharply distinguish between quadrants, and many articles take more than one approach or address more than one EPA element.

## CONCLUSIONS

Emergency medicine is an underrecognized area of entrustable professional activities activity. However, the approach to framework development remains heterogeneous and unstandardized. Small studies address various areas of entrustable professional activity development, including framework creation, trainee and supervisor perceptions, and the use of technology to support entrustable professional activity implementation. Most publications are explorative or translational, highlighting gaps in the experimental research needed to justify the adoption of entrustable professional activities and observational research required to predict real‐world outcomes.

## FUNDING INFORMATION

TB, HB, and VLV are supported by the Rural Health Multidisciplinary Training Program funded by the Australian Government Department of Health and Aged Care.

## CONFLICT OF INTEREST STATEMENT

The authors declare no conflicts of interest.

## Supporting information


Data S1:

Data S2:

## Data Availability

The data supporting this study are derived from publicly available sources, including published literature and publicly accessible databases. Data were extracted and analyzed as part of the review process, but no new primary data were generated.
