# Supplementary material for: Entrustable professional activity use in emergency medicine: A scoping review
Source: AEM Educ Train. 2025 Apr 9;9(2):e70035. doi: 10.1002/aet2.70035 (PMC11982635; doi:10.1002/aet2.70035)
Supplement: Supplementary file 1 — Data S1: [file AET2-9-e70035-s001.docx]

Supplementary Figures

## Table 1S. Entrustable Professional Activities in Emergency Medicine

|  | **Inclusion criteria** | **Exclusion criteria** |
| --- | --- | --- |
| **Participants**  Post-foundation year medical practitioners | Medical practitioners, including postgraduate year one doctors directly entering structured emergency medicine training program (as occurs in the US and Canada) and all post foundation year doctors. | Medical students and foundation-year doctors who have not yet entered structured specialty training (as occurs in the UK, Australia, Denmark, Sweden, Israel, and Japan^32^).  Other clinician groups (such as nurses and paramedics) |
| **Concept**  Entrustable professional activities | Documents using the term ‘entrustable professional activities’ or synonyms to describe a unit of professional practice  *Studies and reports were included even if they involve modifications to the recommended description* | Studies or reports using another method of defining units of medical practice or competence  Studies or reports focusing on competencies, knowledge, skills, or attitudes but not EPAs |
| **Context**  Emergency medicine | Emergency medicine practice performed by emergency medicine, rural generalist, family medicine or similar practitioners  Specific components of EM practice (such as paediatric emergency medicine, resuscitation, or point-of-care ultrasound) Emergency medicine practice performed by emergency medicine, rural generalist, family medicine or similar practitioners  EM trainees also rotate to other specialties (such as mental health), where EM EPAs may be completed  Studies that include emergency medicine training as well as other specialties - if emergency medicine results can be extracted from the results | Practice components of other medical specialties (such as surgery or psychiatry) performed by doctors from those specialties in an emergency department |

Table 1S. This table defines the participants, concept and context that forms the basis of the EPAs in this EM scoping review.

## Table2S: Peer-Review Search Strategy for Entrustable Professional Activities in Emergency Medicine

|  | OVID MEDLINE | EMBASE | CINAHL |
| --- | --- | --- | --- |
| Emergency Medicine | 1. exp Emergency Medicine/ 2. exp Emergency Medical Services/ 3. emergency medic*.mp. 4. emergency servic*.mp. 5. emergency depart*.mp. 6. emergency room*.mp. 7. emergency ward*.mp. 8. emergency physician*.mp. 9. emergency doctor*.mp. 10. 1 or 2 or 3 or 4 or 5 or 6 or 7 or 8 or 9 | 1. ‘emergency medicine’/exp 2. ‘emergency physician’/exp 3. ‘emergency health service’/exp 4. ‘emergency medic*’:ti,ab 5. ‘emergency serv*’:ti,ab 6. ‘emergency department*’:ti,ab 7. ‘emergency room*’:ti,ab 8. ‘emergency ward*’:ti,ab 9. ‘emergency physician*’:ti,ab 10. ‘emergency doctor*’:ti,ab 11. #1 OR #2 OR #3 OR #4 OR #5 OR #6 OR #7 OR #8 OR #9 OR #10 | 1. (MH "Emergency Medicine+") 2. (MH "Physicians, Emergency+") 3. (MH "Emergency Service+") 4. (AB emergency medic* or TI emergency medic*) 5. (AB emergency servic* OR TI emergency servic*) 6. (AB emergency depart* OR TI emergency depart*) 7. (AB emergency room* OR TI emergency room*) 8. (AB emergency ward* or TI emergency ward*) 9. (AB emergency physician* OR TI emergency physician*) 10. (AB emergency doctor* OR TI emergency doctor*) 11. S1 OR S2 OR S3 OR S4 OR S5 OR S6 OR S7 OR S8 OR S9 OR S10 |
| Entrustable professional activities 1 | 1. Entrustable professional*.mp 2. Capabilities in Practice.mp 3. Capability in Practice.mp 4. Observable Practice Activit*.mp 5. 11 or 12 or 13 or 14 | 1. ‘Entrustable Professional activity’/exp 2. ‘entrustable professional*’:ti,ab 3. ‘Capabilities in Practice’ 4. ‘Capability in Practice’:ti,ab 5. ‘Observable Practice Activit*’:ti,ab 6. #12 or #13 or #14 or #15 or #16 | 1. (AB Entrustable professional* OR TI Entrustable professional*) 2. (AB Capabilities in Practice OR TI Capabilities in Practice) 3. (AB Capability in Practice OR TI Capability in Practice) 4. (AB Observable Practice Activit* OR TI Observable Practice Activit*) 5. S12 OR S13 OR S14 OR S15 |
| Entrustable professional activities 2: Abbreviations - linked to Education to remove EPA = Environmental Protection Agency | 1. Entrustable.mp. 2. EPA.mp. 3. EPAs.mp. 4. 16 or 17 or 18 5. 20. Exp Education/ 6. 19 and 20 | 1. ‘Entrustable’:ti,ab 2. ‘EPA’:ti,ab 3. ‘EPAs’:ti,ab 4. #18 OR #19 OR #20 5. ‘education’/exp 6. #21 AND #22 | 1. (AB Entrustable* OR TI Entrustable*) 2. (AB EPA OR TI EPA) 3. (AB EPAs OR TI EPAs) 4. S17 OR S18 OR S19 5. (MH Education+) 6. S20 AND S21 |
| Combine Entrustable Professional Activity term | 1. 15 or 21 | 1. #17 or #23 | 1. S16 AND S22 |
| Combination + limits | 1. 23. 10 and 22 | 1. #11 AND #24 2. #25 AND [embase]/lim NOT ([embase]/lim AND [medline]/lim) | 1. S11 AND S23 |

## Table 3S: Foreign language terms for Entrustable Professional Activities and Emergency Medicine used for Google Advanced Search of the Websites of the International Federation of Emergency Medicine and Member Organisations

| Language | EPA | Emergency Medicine |
| --- | --- | --- |
| French | Activites professionnelles confiables (APC) | Medcin d’urgence |
| Portuguese | Atividades Profissionais Confiáveis |  |
| Spanish | Actividades Profesionales Confiables (APROC) | Medicina de Urgencias |
| German | Anvertraubare professionelle Tätigkeiten (APTs) | Notfall und Akutmedizin |
| Swedish |  | Akutsjukvård, or  Nödfallsmedicin |
| Finnish | Luotettavasti osoitettu pätevyys | Akuuttilääketieteen |
| Greek | Αξιόπιστη Επιστημονική Δραστηριότητα |  |
| Korean | 주창한 위임가능전문직무 |  |
| Hungarian | igazolt szakmai jártasságok |  |
| Lithuanian | Patikėta profesinė veikla |  |
| Russian | доверенной профессиональной активности |  |
| Netherlands |  | Spoedeisende Geneeskunde |
| Danish | betroede professionelle aktiviteter | Akutmedicin |
| Turkish | Güvenilebilir Profesyonel Etkinlik |  |

## Table 4S: Further Details from Included Articles in the Scoping Review of Entrustable Professional Activities (EPAs) in Emergency Medicine

| Reference | Country of Origin | Publication Year | Document Type | Approach | Element | Trainee Focus | Framework Organisation |
| --- | --- | --- | --- | --- | --- | --- | --- |
| Aberdour 2017^39^ | Canada | 2017 | Con. | Explorative | EPA framework | Trainee - training program not specified | Not identified |
| Akbar 2024^40^ | United States | 2024 | Con. | Translational | EPA ecosystem | Trainee - training program not specified | Not identified |
| Alrimawi 2020^41^ | Canada | 2020 | Con. | Experimental | EPA ecosystem | Trainee - training program not specified | RCPSC |
| Beeson 2014A^42^ | United States | 2014 | Con. | Explorative | EPA framework | EM specialty trainees | Independent group of authors |
| Beeson 2014B^43^ | United States | 2014 | Art. | Translational | EPA theory | EM specialty trainees | Not identified |
| Bérczi 2020^44^ | Canada | 2020 | Con. | Translational | EPA program | EM specialty trainees | RCPSC |
| Borman-Shoap 2018^45^ | United States | 2018 | Con. | Experimental | EPA ecosystem | EM specialty trainees | Not identified |
| Boyne 2024^46^ | United States | 2024 | Con. | Translational | EPA ecosystem | EM specialty trainees | Not identified |
| Breckwoldt 2022^47^ | Switzerland | 2022 | Art. | Translational | EPA theory | Consultant - training program not identified | Not identified |
| Caretta-Weyer 2024^48^ | United States | 2024 | Mer. | Explorative | EPA framework | EM specialty trainees | Independent group of authors |
| Carey 2020^49^ | Canada | 2020 | Art. | Explorative | EPA ecosystem | EM specialty trainees | RCPSC |
| CEP Thailand 2019^50^ | Thailand | 2019 | Cur. | Translational | EPA framework | EM specialty trainees | College of Emergency Physicians of Thailand |
| Chan 2020^51^ | Canada | 2020 | Art. | Explorative | EPA program | EM specialty trainees | RCPSC |
| Chang 2023^52^ | Taiwan | 2023 | Art. | Observational | Trainees | EM specialty trainees | Taiwan Society of EM |
| Collings 2019^53^ | Canada | 2019 | Art. | Explorative | Supervisors | EM specialty trainees | RCPSC |
| Costello 2019^54^ | Canada | 2019 | Con. | Explorative | EPA program | EM specialty trainees | RCPSC |
| Costello 2023^55^ | Canada | 2023 | Con. | Explorative | Trainees | EM specialty trainees | RCPSC |
| Denson 2013^38^ | United States | 2013 | Con. | Explorative | EPA framework | Trainee - training program not specified | Independent group of authors |
| Deutscher 2021^56^ | Canada | 2021 | Con. | Explorative | EPA program | EM specialty trainees | RCPSC |
| Fant 2015^57^ | United States | 2015 | Con. | Translational | EPA program | EM specialty trainees | Not identified |
| Fisk 2023^58^ | Canada | 2023 | Con. | Explorative | Supervisors | EM specialty trainees | RCPSC |
| Golden 2021^59^ | United States | 2021 | Con. | Explorative | EPA framework | EM specialty trainees | Council of Residency Directors in EM (proposed list only) |
| Hall 2020^60^ | Canada | 2020 | Mer. | Explorative | EPA program | EM specialty trainees | RCPSC |
| Hart 2019^4^ | United States | 2019 | Art. | Explorative | EPA framework | EM specialty trainees | Independent group of authors |
| Hsiao 2020^61^ | Taiwan | 2020 | Art. | Translational | EPA ecosystem | EM specialty trainees | Not identified |
| Hsu 2016^62^ | United States | 2016 | Art. | Explorative | EPA framework | EM specialty trainees | American Board of Pediatrics |
| Hsu 2023^63^ | United States | 2023 | Art. | Observational | Supervisors | EM specialty trainees | American Board of Pediatrics |
| Jaber 2024^64^ | United States | 2024 | Con. | Explorative | Trainees | EM specialty trainees | Not identified |
| Koh 2019^65^ | Canada | 2019 | Art. | Translational | EPA theory | EM specialty trainees | RCPSC |
| Lai 2019^66^ | United States | 2019 | Con. | Observational | Supervisors | EM specialty trainees | Not identified |
| Landreville 2022^67^ | Canada | 2022 | Art. | Observational | Supervisors | EM specialty trainees | RCPSC |
| Lee 2020^68^ | Taiwan | 2020 | Art. | Explorative | EPA ecosystem | EM specialty trainees | Not identified |
| Lee 2021^69^ | Taiwan | 2021 | Art. | Explorative | Supervisors | EM specialty trainees | Not identified |
| Lui 2023^70^ | Hong Kong | 2023 | Cur. | Translational | EPA framework | EM specialty trainees | Hong Kong College of EM |
| Pandya 2022^71^ | Canada | 2022 | Art. | Translational | Trainees | EM specialty trainees | RCPSC |
| Paterson 2023^72^ | Canada | 2023 | Art. | Explorative | Supervisors | EM specialty trainees | RCPSC |
| Prudhomme 2020^73^ | Canada | 2020 | Art. | Observational | Trainees | EM specialty trainees | RCPSC |
| RCPSC 2017^74^ | Canada | 2018 | Cur. | Translational | EPA framework | EM specialty trainees | RCPSC |
| Sagalowsky 2023^75^ | United States | 2023 | Art. | Translational | EPA theory | EM specialty trainees | American Board of Pediatrics |
| Sahi 2024^76^ | Canada | 2024 | Art. | Explorative | Trainees | EM specialty trainees | RCPSC |
| Sample 2021^77^ | Canada | 2021 | Art. | Translational | EPA ecosystem | EM specialty trainees | RCPSC |
| Saraburi 2018^78^ | Thailand | 2018 | Cur. | Translational | EPA framework | Trainee - training program not specified | Thai Ministry of Public Health |
| Seed 2023^79^ | Canada | 2023 | Art. | Observational | Trainees | EM specialty trainees | RCPSC |
| Sherbino 2020^80^ | Canada | 2020 | Art. | Translational | EPA theory | EM specialty trainees | RCPSC |
| Singh 2023^81^ | Canada | 2023 | Mer. | Observational | Supervisors | EM specialty trainees | RCPSC |
| Spadafore 2024^82^ | Canada | 2024 | Mer. | Explorative | Supervisors | EM specialty trainees | RCPSC |
| Stanford PEM 2016 | United States | 2016 | Cur. | Translational | EPA framework | EM specialty trainees | Stanford University PEM Program |
| Stoneham 2019^83^ | Canada | 2019 | Mer. | Translational | EPA ecosystem | EM specialty trainees | RCPSC |
| Taiwan SEM 2019^84^ | Taiwan | 2019 | Cur. | Translational | EPA framework | EM specialty trainees | Taiwan Society of EM |
| Thoma 2020^85^ | Canada | 2020 | Mer. | Observational | EPA program | EM specialty trainees | RCPSC |
| Tiyyagura 2014^86^ | United States | 2014 | Art. | Explorative | Supervisors | EM specialty trainees | American Board of Pediatrics |
| Turner 2021^87^ | United States | 2021 | Art. | Observational | Supervisors | EM specialty trainees | American Board of Pediatrics |
| Villa 2024^88^ | United States | 2024 | Art. | Explorative | EPA framework | Trainee - training program not specified | Independent group of authors |
| Vincent 2023^89^ | Canada | 2023 | Con. | Translational | EPA ecosystem | EM specialty trainees | RCPSC |
| Woods 2022^90^ | Canada | 2022 | Art. | Explorative | Supervisors | EM specialty trainees | RCPSC |
| Woods 2023^91^ | Canada | 2023 | Con. | Explorative | Supervisors | EM specialty trainees | RCPSC |
| Yilmaz 2022^92^ | Canada | 2021 | Mer. | Translational | Supervisors | EM specialty trainees | RCPSC |
| Yilmaz 2023^93^ | Canada | 2022 | Art. | Translational | EPA ecosystem | EM specialty trainees | RCPSC |

This table summarizes further characteristics of the studies included in the scoping review of EPAs in emergency medicine, detailing their article title, specific methods, assessment focus, document type and whether an EPA framework was included or identified. *Art.* journal article, *Con.* conference proceedings, *Mer.* Journal article merged with one or more conference proceedings, *Cur.* Curriculum document. *RCPSC* Royal College of Physicians and Surgeons of Canada

## Table 5S: Entrustable Professional Activity Titles from Included Articles

| **Reference** | **EPA topic focus** | **Organisation** | **EPA Titles** |
| --- | --- | --- | --- |
| Hsu 2016 | Pediatric EM | American Board of Pediatrics | - Provide for and obtain consultation from other health care providers caring for children - Contribute to the fiscally sound and ethical management of a practice (e.g., through billing, scheduling, coding, and record keeping practices) - Apply public health principles and improvement methodology to improve care for populations, communities, and systems - Lead an interprofessional health care team - Facilitate handovers to another health care provider - Engage in scholarly activities through the discovery, application, and dissemination of new knowledge - Lead within the subspecialty profession - Recognize and provide care for acutely ill and/or injured pediatric patients presenting to the emergency department - Recognize and provide care for medically and technologically complex pediatric patients in the ED - Demonstrate competence in performing common procedures associated with the practice of pediatric emergency medicine - Provide patient triage, resuscitation, and stabilization; align care provided with severity of illness - Emergency department management: Manage the emergency department to optimize patient care - Provide supervision for emergency personnel to enhance patient care quality and assure patient safety |
| Stanford PEM 2016 | Pediatric EM | Stanford University PEM Program | - Recognize and provide care for acutely ill and/or injured pediatric patients presenting to the Emergency Department (ED) - Recognize and provide care for medically and technologically complex pediatric patients in the ED - Demonstrate competence in performing common procedures associated with the practice of pediatric emergency medicine - Provide patient triage, resuscitation, and stabilization; align care provided with severity of illness. - Emergency Department Management: Manage the emergency department to optimize patient care - Provide supervision for emergency personnel to enhance patient care quality and assure patient safety. |
| Denson 2013 | Geriatric EM | Independent group of authors | - Identify delirium using cognitive exam - Discuss available home care services |
| Beeson 2014A | General EM | Independent group of authors | - Ankle injury - Wrist injury - Chest pain - Pharyngitis - Altered mental status - Back pain - Headache - Abdominal pain - Shortness of breath - Syncope |
| Beeson 2014B | General EM | Not identified | - Altered mental status - Back pain - Headache - Multiple trauma - Abdominal pain - Pelvic pain - Vaginal bleeding - Chest pain - Shortness of breath - Cough - Eye disorders - ENT [ear, nose, and throat] complaints - Rash - Ankle/wrist injury - Pediatric respiratory distress - Pediatric dehydration - Depression - The critical patient (by vital signs, respiratory failure, etc.) - Delivering bad news - Difficult patient/family/consultant interaction - General procedures - Airway management - Wound care - Ultrasound POC [point of care] - Vascular access - Anesthesia and pain management |
| Caretta-Weyer 2024 | General EM | Independent group of authors | - Initiate treatment for a patient requiring emergent/immediate intervention. - Lead the resuscitation of a critically ill or injured patient. - Obtain and interpret a focused history using data from all necessary sources. - Perform and interpret a focused physical examination. - Create and prioritize a differential diagnosis. - Order and interpret diagnostic tests. - Apply best available evidence to guide patient care. - Manage clinical or diagnostic uncertainty when caring for patients. - Utilize observation and reassessment to guide decision making. - Develop and implement an appropriate disposition and aftercare plan. - Perform the diagnostic and therapeutic procedures of an emergency physician. - Provide invasive and noninvasive airway management. - Perform and interpret point-of-care ultrasound. - Perform procedural sedation. - Implement pharmacologic and therapeutic management plans. - Provide palliative and end-of-life care for patients and their families. - Document the ED encounter. - Communicate with other health care professionals about patient care. - Communicate with the patient, family, and caregivers. - Provide supervision or consultation for other health care professionals. - Manage the ED flow to optimize patient care. - Fulfill professional obligations and adhere to professional standards. |
| CEP Thailand 2019 | General EM | College of Emergency Physicians of Thailand | - Emergency Care - Academic presentations and improving the quality of patient care - Recording medical records - Procedure skills: rapid sequence induction, Central venous catheter insertion, Point of Care Ultrasound, cardiopulmonary resuscitation team leader |
| Hart 2019 | General EM | Independent group of authors | - Manage a low-acuity, low-complexity ‘stable’ patient. - Manage a low-acuity, high-complexity ‘stable’ patient - Manage a potentially high-acuity complaint in a ‘stable’ patient - Manage a high-acuity patient with a well-defined presentation, illness, or injury - Manage a high-acuity, high-complexity patient (i.e., the undifferentiated unstable patient) - Manage multiple patients in the emergency department (ED) concomitantly - Lead an ED team - Transition patient care to other healthcare providers - Manage interactions with consultants - Manage complex and difficult situations - Use recommended patient-safety and quality improvement processes |
| Lui 2023 | General EM | Hong Kong College of Emergency Medicine | - Rapid Sequence Intubation - Chest Drain / Needle aspiration - Closed reduction of Shoulder dislocation - Closed reduction of Colles fracture |
| RCPSC 2018 | General EM | Royal College of Physicians and Surgeons of Canada | - TD 1: Recognizing the unstable/critically ill patient, mobilizing the healthcare team and supervisor, and initiating basic life support - TD 2: Performing and documenting focused histories and physical exams, and providing preliminary management of cardinal emergency department presentations - TD 3: Facilitating communication of information between a patient in the emergency department, caregivers, and members of the healthcare team to organize care and disposition of the patient - F1: Initiating and assisting in resuscitation of critically ill patients - F2: Assessing and managing patients with uncomplicated urgent and non-urgent emergency department presentations - F3: Contributing to the shared work of the emergency department health care team to achieve high quality, efficient and safe patient care - F4: Performing basic procedures - C1: Resuscitating and coordinating care for critically ill patients - C2: Resuscitating and coordinating care for critically injured trauma patients - C3: Providing airway management and ventilation - C4: Providing emergency sedation and systemic analgesia for diagnostic and therapeutic procedures - C5: Identifying and managing patients with emergent medical or surgical conditions - C6: Diagnosing and managing patients with complicated urgent and non-urgent patient presentations - C7: Managing urgent and emergent presentations for pregnant and post-partum patients - C8: Managing patients with acute toxic ingestion or exposure - C9: Managing patients with emergency mental health conditions or behavioural emergencies - C10: Managing and supporting patients in situational crisis to access health care and community resources - C11: Recognizing and managing patients who are at risk of exposure to, or who have experienced violence and/or neglect - C12: Liaising with prehospital emergency medical services - C13: Performing advanced procedures - C14: Performing and interpreting point-of-care ultrasound to guide patient management - C15: Providing end-of-life care for a patient - TP1: Managing the emergency department to optimize patient care and department flow - TP2: Teaching and supervising the learning of trainees and other health care professionals - TP3: Managing complex interpersonal interactions that arise during the course of patient care - TP4: Providing expert EM consultation to physicians or other healthcare providers - TP5: Coordinating and collaborating with healthcare professional colleagues to safely transition the care of patients, including handover and facilitating inter-institution transport - TP6: Dealing with uncertainty when managing patients with ambiguous presentations |
| Saraburi 2018 | General EM | Thai Ministry of Public Health | - General Emergency Care - Academic Presentations and Improving the Quality of Patient Care - Medical Record Recording - Cardiopulmonary resuscitation: Cardiopulmonary resuscitation - Point of care ultrasound: Point of Care Ultrasound - Septic Shock Management - Rapid sequence intubation: rapid sequence induction - Multiple Trauma Management - Central Venous Access - Procedural Sedation |
| Taiwan SEM 2019 | General EM | Taiwan Society of Emergency Medicine | - Treat patients with cardiac arrest before arriving at the hospital - Disposition of patients in shock - Disposition of patients with major external injuries - Disposition of poisoned patients - Acute chest pain patient management - Treatment of patients with acute consciousness change - Treatment of patients with acute respiratory difficulties |
| Villa 2024 | EM education | Independent group of authors | - Creating an academic curriculum vitae - Creating an educator portfolio - Serving as a mentor to learners - Performing a needs assessment - Creating curricula incorporating elements of education theory - Applying evidence-based teaching methods to didactic instruction - Utilizing a variety of bedside teaching techniques - Applying educational methods that foster diversity, equity, and inclusion - Evaluating curricula - Providing feedback to learners - Creating an individualized learning plan to support a struggling learner - Designing a scholarly education project - Assessing quality of Medical Education research - Performing a program evaluation - Performing workplace-based learner assessments - Participating in education committees |

This table summarizes the topic focus, entrustable professional activity titles and the organization creating or administering the EPA framework of EPA frameworks included in the scoping review
