# Supplementary material for: Entrustable professional activity use in emergency medicine: A scoping review
Source: AEM Educ Train. 2025 Apr 9;9(2):e70035. doi: 10.1002/aet2.70035 (PMC11982635; doi:10.1002/aet2.70035)
Supplement: Supplementary file 2 — Data S2: [file AET2-9-e70035-s002.docx]

Supplementary Material:

Data Extraction Tool for Scoping Review (Covidence)

This document is a copy of the data tool used in Covidence

**General Information**

- **Study ID:**
- **First Author Name and Year of Publication:**
- **Document Title:** (Title of paper/abstract/report from which data are extracted)
- **Country of Origin:**

☐ Canada

☐ United States

☐ UK

☐ Taiwan

☐ Australia

☐ Other

- **Publication Year:**
- **Notes:**

**Characteristics of Included Studies**

**Aim / Purpose**

- **Article Approach:**

☐ **Explorative** (Modelling and describing underlying characteristics)

☐ **Experimental** (Testing effects of interventions)

☐ **Observational** (Examining relationships between variables)

☐ **Translational** (Implementing research into real-world settings)

*Ref: Ringsted C, Hodges B, Scherpbier A. ‘The Research Compass’: An introduction to research in medical education. Med Teach. 2011;33(9):695-709.*

- **Specific Methods:**

**Participants**

- **Doctors who are undergoing or have completed specialty training:**

☐ EM Specialty Trainees

☐ EM Consultants

☐ Family Medicine/Generalist Trainees

☐ Family Medicine/Generalist Consultants

☐ Trainee – Training Program Not Specified

☐ Consultant – Training Program Not Identified

☐ Not Specified

☐ Other

- **Context: Emergency Medicine or Subspecialty:**

☐ General Emergency Medicine

☐ Paediatric Emergency Medicine

☐ Resuscitation in Emergency Medicine

☐ Ultrasound in Emergency Medicine

☐ Education in Emergency Medicine

☐ Rural Emergency Medicine

☐ Other

**Concept: Entrustable Professional Activities (EPAs)**

- **EPA Element:**

☐ **EPA Theory** (Addresses theoretical foundations and relation to competence-based training)

☐ **EPA Framework** (Describes EPA structure, components, and required competencies)

☐ **EPA Program** (Macro-level, evaluating system-wide implementation and effects)

☐ **EPA Ecosystem** (Meso-level, administration of assessment and information flow)

☐ **Trainees** (Micro-level, methods of assessment and attitudes)

☐ **Supervisors** (Micro-level, supervisor feedback and expectations)

☐ **Other**

- **EPA Framework Identification:**

☐ Described in Article or Supplementary Material

☐ Identified in Article and Reference Provided

☐ Identified in Article but No Reference Provided

☐ Not Identified

☐ Other

- **EPA Framework Reference (If cited):**
- **Organisation Creating/Administering EPA Framework:**

☐ Royal College of Physicians and Surgeons of Canada

☐ American Board of Paediatrics

☐ Taiwan Society of Emergency Medicine

☐ Independent Group of Authors

☐ Not Identified

☐ Other

- **EPA Framework Description:**
  - **Has the EPA Framework already been extracted from another document?**

☐ Yes

☐ No

☐ Other

- - **If yes, what is the Study ID of the document from which the EPA Framework was previously extracted?**
  - **If no, list the titles of the EPAs.**

**Logic Behind EPA Titles**

- **Service Provision** *(e.g., Arranging patient transfer, Supervising junior staff)*
- **Procedures** *(e.g., Performing critical care procedures, Intubation)*
- **Disease and Patient Groups** *(e.g., Managing shock, Undifferentiated illness)*

*Ref: Hennus MP, van Dam M, Gauthier S, Taylor DR, Ten Cate O. The logic behind entrustable professional activity frameworks: A scoping review of the literature. Med Educ. 2022;56(9):881-91.*

**Number of EPAs Identified**

| **Category** | **Count** |
| --- | --- |
| Service Provision |  |
| Procedures |  |
| Diseases/Patient Groups |  |
| Uncertain |  |
| **Total EPAs** |  |
